# Coding long COVID: characterizing a new disease through an ICD-10 lens

**DOI:** 10.1186/s12916-023-02737-6

**Published:** 2023-02-16

**Authors:** Emily R. Pfaff, Charisse Madlock-Brown, John M. Baratta, Abhishek Bhatia, Hannah Davis, Andrew Girvin, Elaine Hill, Elizabeth Kelly, Kristin Kostka, Johanna Loomba, Julie A. McMurry, Rachel Wong, Tellen D. Bennett, Richard Moffitt, Christopher G. Chute, Melissa Haendel, The N3C Consortium, The RECOVER Consortium

**Affiliations:** 1 University of North Carolina at Chapel Hill, Chapel Hill, USA; 2 University of Tennessee Health Science Center, Memphis, USA; 3 Patient-Led Research Collaborative, New York, USA; 4 Palantir Technologies, Denver, USA; 5 University of Rochester, Rochester, USA; 6 Northeastern University, Boston, USA; 7 University of Virginia, Charlottesville, USA; 8 University of Colorado Anschutz Medical Campus, Aurora, USA; 9 Stony Brook University, Stony Brook, USA; 10 Johns Hopkins University, Baltimore, USA

**Keywords:** Long COVID, Electronic health records, Health disparities

## Abstract

**Background:**

Naming a newly discovered disease is a difficult process; in the context of the COVID-19 pandemic and the existence of post-acute sequelae of SARS-CoV-2 infection (PASC), which includes long COVID, it has proven especially challenging. Disease definitions and assignment of a diagnosis code are often asynchronous and iterative. The clinical definition and our understanding of the underlying mechanisms of long COVID are still in flux, and the deployment of an ICD-10-CM code for long COVID in the USA took nearly 2 years after patients had begun to describe their condition. Here, we leverage the largest publicly available HIPAA-limited dataset about patients with COVID-19 in the US to examine the heterogeneity of adoption and use of U09.9, the ICD-10-CM code for “Post COVID-19 condition, unspecified.”

**Methods:**

We undertook a number of analyses to characterize the N3C population with a U09.9 diagnosis code (*n* = 33,782), including assessing person-level demographics and a number of area-level social determinants of health; diagnoses commonly co-occurring with U09.9, clustered using the Louvain algorithm; and quantifying medications and procedures recorded within 60 days of U09.9 diagnosis. We stratified all analyses by age group in order to discern differing patterns of care across the lifespan.

**Results:**

We established the diagnoses most commonly co-occurring with U09.9 and algorithmically clustered them into four major categories: cardiopulmonary, neurological, gastrointestinal, and comorbid conditions. Importantly, we discovered that the population of patients diagnosed with U09.9 is demographically skewed toward female, White, non-Hispanic individuals, as well as individuals living in areas with low poverty and low unemployment. Our results also include a characterization of common procedures and medications associated with U09.9-coded patients.

**Conclusions:**

This work offers insight into potential subtypes and current practice patterns around long COVID and speaks to the existence of disparities in the diagnosis of patients with long COVID. This latter finding in particular requires further research and urgent remediation.

**Supplementary Information:**

The online version contains supplementary material available at 10.1186/s12916-023-02737-6.

## Background

Naming diseases is an ever-present challenge, and there is no shortage of efforts that aim to better standardize, disambiguate, and keep track of disease nomenclature and definitions [1–4]. Disease naming has long been controversial–for example, there are more than 400 names for syphilis dating back to the fifteenth century [5]. Naming a disease requires defining it, and assigning a standard code to the disease facilitates research, care, and patient engagement due to the ease of patient classification and knowledge exchange. However, naming and coding a disease does not mean the disease did not exist prior to its naming or coding. For instance, although “SARS-CoV-2” and “COVID-19” were both coined on February 11, 2020, by the International Committee on the Taxonomy of Viruses and the World Health Organization (WHO), respectively [6, 7], we know that cases of COVID-19 began to surface in Wuhan, China in late December 2019 [8]. In the USA, most diagnostic coding uses the International Classification of Diseases 10th edition, clinical modification (ICD-10-CM) terminology; however, the ICD-10-CM code for COVID-19, U07.1, was not made available for use until April 1, 2020. The implications of this naming delay are wide-ranging. To this day, US COVID-19 cases prior to April 1, 2020, are difficult to retrospectively ascertain. Even after that date, use of U07.1 for COVID-19 phenotyping came with caveats–use of the new code was inconsistent and of variable sensitivity and specificity, and studies have shown both underuse and overuse of U07.1 in different contexts and health systems [9–11].

Long COVID, which is included in the more general term of post-acute sequelae of SARS CoV-2 infection (PASC), is also subject to the effects of delayed naming. By Spring of 2020, patients suffering from long COVID had coined various terms to describe the condition, including the COVID-19 long tail, long-haul COVID, and long COVID [12–14]. Long COVID is defined by ongoing, relapsing, or new symptoms or other health effects occurring after the acute phase of SARS-CoV-2 infection (i.e., present four or more weeks after the acute infection). Heterogeneous symptoms may include, but are not limited to, fatigue, difficulty breathing, brain fog, insomnia, joint pain, and cardiac issues [15–17]. As the impact of long COVID on health and quality of life became increasingly clear at a population level, patients worldwide came together to urge healthcare systems and policymakers to acknowledge this condition [18, 19].

Despite the relatively early recognition of this condition, an ICD-10-CM code (U09.9, “Post COVID-19 condition, unspecified”) was not made available for use in the clinical setting until October 2021. Moreover, this single code may prove insufficient: considering the phenotypic and severity variation seen in long COVID patients, it is likely that subtypes of long COVID exist, and such subtypes may correlate with specific underlying mechanisms that should be targeted by different interventions.

Regardless, the fact remains that there is more naming to be done, and a particular need to define and refine computable phenotypes for long COVID and its subtypes. As can be seen by the widely differing estimates of long COVID prevalence across many studies, a lack of definitional consistency is affecting the accuracy and reproducibility of otherwise robust research [20]. Among other advantages, refined definitions will enable us to appropriately define cohorts for clinical studies, provide more precise treatment and clinical decision support, and accurately estimate long COVID’s incidence and prevalence. This is a key priority for the parent program for this work, the NIH Researching COVID to Enhance Recovery (RECOVER) Initiative [21], which seeks to understand, treat, and prevent PASC through a wide variety of research modalities, including electronic health record (EHR) and real-world data.

In response to the COVID-19 pandemic, the US informatics and clinical community harmonized an enormous amount of EHR data to reveal candidate risk factors and therapies associated with COVID-19. The National Institute of Health’s (NIH) National COVID Cohort Collaborative (N3C) is now the largest publicly available Health Insurance Portability and Accountability Act (HIPAA) limited EHR dataset in U.S. history, with over 16 million patients. Due to the scale and demographic and geographic diversity of data within the N3C, it is uniquely well-suited to characterize the early use of the new long COVID ICD-10-CM code. Here, we seek to characterize both (1) the early clinical use patterns of U09.9 and (2) the patients receiving that code from a provider. These characterizations reveal interesting patterns that may enable us to glean a better understanding of rough subtypes of long COVID, current clinical practices for diagnosis and treatment of long COVID, and potential racial and social disparities affecting who seeks and receives care for long COVID. Ultimately, identifying patients with long COVID based upon multiple means of inquiry (including U09.9) is critically important to recruit participants for research studies, assess the public health burden, and support nimble analytics across our heterogenous health care systems.

## Methods

To characterize the use of the U09.9 code, we used EHR data integrated and harmonized inside the NIH-hosted N3C Secure Data Enclave to identify clinical features co-occurring around the time of patients’ U09.9 index date. The methods for patient identification, data acquisition, ingestion, and harmonization into the N3C Enclave have been described previously [22–24]. Briefly, N3C contains EHR data for patients (1) who tested positive for SARS-CoV-2 infection; (2) who have a diagnosis code for COVID-19 (U07.1), multisystem inflammatory syndrome (MIS-C, M35.81), or long COVID (U09.9); (3) whose symptoms are consistent with a COVID-19 diagnosis; or (4) are demographically matched controls who have tested negative for SARS-CoV-2 infection (and have never tested or been diagnosed as positive) to support comparative studies. Lookback data are available from January 2018 forward for each patient.

In this retrospective cohort study, we defined our initial population (*n* = 36,880, sourced from 34 different health care systems) as any non-deceased patient with one or more U09.9 diagnosis codes recorded between October 1, 2021, and May 26, 2022. U09.9 codes appearing prior to October 1, 2021, may have been retroactively applied to these patients’ records (e.g., as “onset dates” in an EHR Problem List), therefore making it difficult to determine an index date that reflects the actual date of diagnosis. We excluded patients (*n* = 3098) whose U09.9 index occurred during an inpatient hospitalization, due to the difficulty of distinguishing co-occurring clinical features related to long COVID versus the primary reason for their hospitalization. After these exclusions, a base population of 33,782 remained. Note that we did not require patients in our cohort to have a COVID-19 diagnosis code (U07.1) or positive SARS-CoV-2 test on record, as many patients with long COVID do not have this documentation [19]. This lack of documentation will only increase over time with the rise of at-home testing.

It should be noted that in a large, harmonized dataset such as N3C, we are not able to differentiate *clinical* diagnosis codes (i.e., a code entered by a provider to signify “this patient has this disease,” often as part of an EHR Problem List) and *billing* diagnosis codes (i.e., a code entered by a provider or medical coder to support billing or insurance reimbursement for the visit) with high certainty. Use of the U09.9 code in both contexts is important to examine; in this analysis, those contexts are combined.

Data from 34 of the 76 N3C sites were used for this analysis. The remaining sites either (1) did not use the U09.9 code in their N3C data or had not refreshed data since November 1, 2021, meaning the U09.9 code would not be present even if used at the site (*n* = 21 sites); (2) had location data missing for all patients (*n* = 12 sites); or (3) did not meet the minimum criteria we set for site data for all RECOVER-related analyses (*n* = 9 sites): (a) >  = 25% of inpatients with at least one white blood cell count and at least one serum creatinine (to ensure lab measurement completeness); (b) 75% of inpatient visits have valid end dates; and (c) dates must not be shifted by the site more than 30 days. Additional N3C data quality criteria have been described previously, and also apply to this work; a summary of these checks is included in Additional file 1: Supplemental Methods [23]. The 34 sites used here are diverse in geographic location and institution size, but cannot be specifically named due to N3C governance policies. Though the N3C data are harmonized, there is variation in the timing of U09.9 uptake among the 34 sites. A visualization of these temporal differences is included as Additional file 1: Supplemental Fig. 1.

**Fig. 1:**
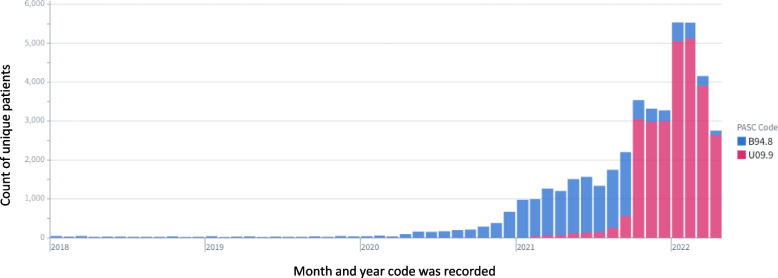
Clinical use of B94.8 decreases as U09.9 becomes available. Prior to U09.9’s release, the CDC recommended use of B94.8 (“Sequelae of other specified infectious and parasitic diseases”) as a placeholder code to signify long COVID. As this code is not specific to sequelae of COVID-19, this figure shows consistent but infrequent use during two pre-pandemic years. Use of B94.8 ramps up in Spring of 2020, suggesting increased recognition of long COVID by providers. However, upon its release in October 2021, U09.9 supplants B94.8 in terms of usage frequency

We calculated person-level demographics and a number of social determinants of health (SDoH) variables at the area level. These variables are sourced from the American Community Survey (ACS) 2019 5-year estimates at the ZIP Code Tabulation Area (ZCTA) level [25]. ZCTAs were mapped to the patients’ 5-digit ZIP code using the USD mapper [26]. SDoH variables were categorized into high, medium, and low based on percentiles for all ZIP codes in the ACS dataset. We then characterized this cohort by examining diagnoses, procedures, and medications that occurred between each patient’s U09.9 index date and 60 days after index (hereafter referred to as our “analysis window”).

### Diagnosis analysis

Our objective in characterizing diagnoses around the U09.9 index date was not only to catalog conditions and symptoms that tend to co-occur with the U09.9 diagnosis, but also to determine which of those conditions and symptoms tend to co-occur with each other. In doing so, we begin to see clusters of conditions that are more likely to occur together within a single patient’s record. First, we extracted all conditions in each patient’s record within the analysis window and identified the most frequently occurring conditions in the study population. We did *not* exclude conditions if they were also reported prior to the patient’s U09.9 index date. Excluding such conditions would discount the possibility that pre-existing conditions could be exacerbated by long COVID, or that long COVID could be associated with new, unrelated instances of prior conditions (e.g., abdominal pain). Because this level of detail is not knowable, our clusters are intended to represent co-occurrence with long COVID, and cannot be interpreted as causation by long COVID.

We then constructed an adjacency matrix for the top 30 conditions, with values indicating the frequency of co-occurrence between two conditions in the study population. From this matrix, we constructed a weighted network with nodes representing individual diagnoses, edges between nodes representing co-occurrence, and edge weights corresponding to the count of patients with both conditions. In order to detect conditions that are more likely to co-occur in our study population than at random, we tested the Louvain [27], Walktrap [28], and Girvan-Newman [29] algorithms for community detection. We selected the Louvain algorithm in our final model, as it maximized modularity while retaining a reasonable resolution of detection. For further subgroup analyses, we present clusters detected within age-stratified condition co-occurrence networks. Additional details on community detection, network stability, and subgroup analyses are available in Additional file 1: Supplemental Methods.

### Procedure analysis

Characterizing common procedures around the time of U09.9 allowed us to assess current practice patterns (i.e., diagnostics and treatments) for patients receiving the code. We defined a “procedure” as any medical diagnostics or treatments rendered by a healthcare provider. We excluded non-informative records that simply reflect that an encounter took place (e.g., Current Procedure Terminology (CPT) 99212, “Office or other outpatient visit”), despite their technical classification as “procedure codes.” We then aggregated the remaining procedures into high-level categories (e.g., “radiography,” “physical therapy”) in order to discern the diagnostics and treatments that occurred within each patient’s analysis window.

### Medication analysis

As with diagnoses and procedures, we extracted all medication records occurring within each patient’s analysis window, in order to characterize newly prescribed medications that may be used to treat symptoms of long COVID. In order to focus on newly prescribed medications and not long-standing prescriptions, we excluded medications for each patient for which there were records prior to the patient’s U09.9 index. Medications were categorized using the third level of the Anatomical Therapeutic Chemical (ATC) classification system [30]. Results of this analysis are shown in Additional file 1: Supplemental Fig. 2.

**Fig. 2:**
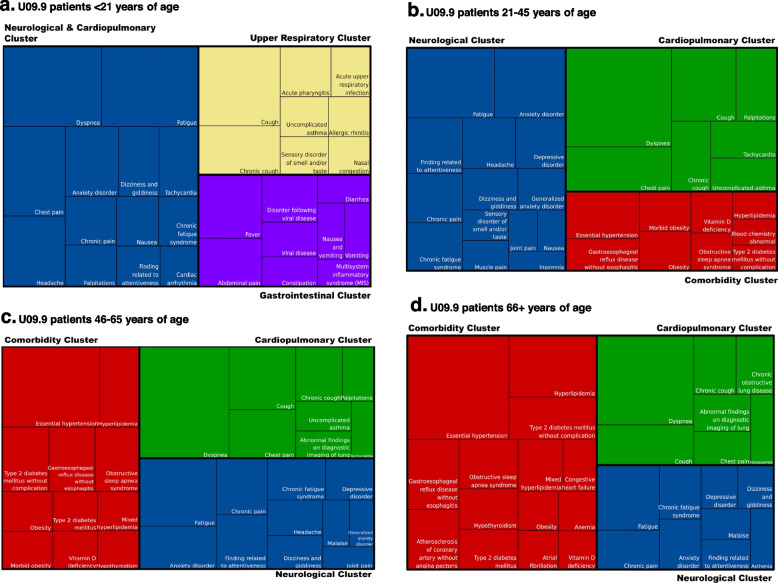
Age-stratified clusters of co-occurring diagnoses among patients with a U09.9 code. When the Louvain algorithm is applied to the top 30 most frequent pairs of co-occurring diagnoses for U09.9 patients (i.e., diagnoses co-occurring in the same patient 0 through 60 days from U09.9 diagnosis date), distinct clusters emerge. These clusters may represent rough subtypes of long COVID presentations, and differ among age groups. The size of each box within a cluster reflects the frequency of that diagnosis relative to others in the diagram. Condition names are derived from the SNOMED CT terminology, mapped from their ICD-10-CM equivalents. Similar clusters share the same color across all four diagrams. **a** U09.9 patients < 21 years of age. **b** U09.9 patients 21–45 years of age. **c** U09.9 patients 46–65 years of age. **d** U09.9 patients 66 + years of age

## Results

Greater severity of acute SARS-CoV-2 infection does not appear to have an outsize influence in determining which patients end up with a U09.9 code; 3266 of the U09.9 patients (9.7%) were hospitalized during a prior acute SARS-CoV-2 infection. This proportion of hospitalized patients is markedly lower than that cited in a recent FAIR Health white paper, which noted that 25% of patients with a U09.9 code recorded in claims data had been hospitalized with acute COVID-19 [31]. Of the patients hospitalized with acute COVID-19, 791 (2.3% of all U09.9 patients, 24.2% of U09.9 patients hospitalized) had hospitalizations categorized as “severe,” with recorded use of a ventilator, extracorporeal membrane oxygenation (ECMO), or vasopressors. Also notable is the fact that 12,550 (37.2%) of the U09.9 patients did not have a COVID index date available in N3C’s records, suggesting that these patients’ acute SARS-CoV-2 infection was indicated by a test at home, at an external health care system, or at a testing site not connected to a health system (e.g., drugstore, airport, workplace). Table 1 shows the breakdown of the study cohort by person-level demographics and area-level social determinants of health. An equivalent breakdown of all SARS-CoV-2 positive patients from the same 34 N3C sites is available as Additional file 1: Supplemental Table 1.

**Table 1:** Demographic breakdown of patients in N3C with a U09.9 diagnosis code. In addition to person-level demographics, we have included a number of social determinants of health variables at the *area* level (see the “Methods” section). In accordance with the N3C download policy, for demographics where small cell sizes (< 20 patients) could be derived from context, we have shifted the counts + / − by a random number between 1 and 5. The accompanying percentages reflect the shifted number. All shifted counts are labeled as such, e.g. + / − 5

	**Age < 21**	**21–45**	**46–65**	**66 + **
	***n*** ** = 2316**	***n*** ** = 11,364**	***n*** ** = 13,850**	***n*** ** = 6252**
**Person-level variables**				
**Sex (%)**				
**Female**	1319 (57.0)	8298 (73.0) + / − 5	9244 (66.7)	3760 (60.1)
**Male**	997 (43.0) + / − 5	3066 (26.9) + / − 5	4606 (33.2) + / − 5	2492 (39.9)
**Unknown**	< 20	< 20	< 20	< 20
**Race (%)**				
**American Indian or Alaska Native**	< 20	112 (1.0)	133 (1.0)	42 (0.7)
**Asian**	57 (2.4) + / − 5	303 (2.7)	257 (1.9)	96 (1.5) + / − 5
**Black**	349 (14.9) + / − 5	1703 (14.9)	1998 (14.4) + / − 5	621 (9.9) + / − 5
**Hawaiian/Pac Isldr**	< 20	23 (0.2)	< 20	< 20
**White**	1516 (65.0) + / − 5	7691 (67.7)	10,022 (72.3) + / − 5	5064 (80.8) + / − 5
**Other**	66 (2.8)	62 (0.5)	52 (0.4) + / − 5	< 20
**Unknown**	328 (14.2)	1469 (12.9)	1387 (10.0)	429 (6.9)
**Ethnicity (%)**				
**Hispanic/Latino**	363 (15.7)	1368 (12.0)	1332 (9.6)	335 (5.4)
**Not Hispanic/Latino**	1687 (72.8)	8785 (77.3)	11,053 (79.8	5355 (85.7)
**Unknown**	266 (11.5)	1211 (10.7)	1465 (10.6)	562 (9.0)
**Area-level social determinants of health (ZIP-code level)**				
**Households with income below poverty (%)**				
**High (> 15.30%)**	604 (26.1)	3288 (28.9)	4098 (29.6)	1832 (29.3)
**Medium (7.92–15.30%)**	691 (29.8)	3624 (31.9)	4295 (31.0)	2008 (32.1)
**Low (< 7.92%)**	790 (34.1)	3265 (28.7)	4089 (29.5)	1888 (30.2)
**Missing**	231(10.0)	1187 (10.4)	1368 (9.9)	524 (8.4)
**Residents with college degree (%)**				
**High (> 17.54%)**	1298 (56.0)	6169 (54.3)	6975 (50.4)	3309 (52.9)
**Medium (10.27–17.54%)**	575 (24.8)	2812(24.7)	3742 (27.0)	1669 (26.7)
**Low (< 10.27%)**	215 (9.3)	1197 (10.5	1765 (12.7)	751 (12.0)
**Missing**	228 (9.8)	1186 (10.4)	1368 (9.9)	523 (8.4)
**Residents 19–64 with public health insurance (%)**				
**High (> 22.50%)**	442 (19.1)	2249 (19.8)	2959 (21.4)	1343 (21.5)
**Medium (12.99–22.50%)**	734 (31.7)	3652 (32.1)	4607 (33.3)	2132 (34.1)
**Low (< 12.99%)**	912 (39.4)	4275 (37.6)	4915 (35.5)	2253 (36.0)
**Missing**	228 (9.8)	1188 (10.5)	1369 (9.9)	524 (8.4)
**Residents 19–64 unemployed (%)**				
**High (> 5.7%)**	555 (23.8)	2870 (25.3)	3789 (27.4)	1655 (26.5)
**Medium (3.1–5.7%)**	1102 (47.6)	5132 (45.2)	6068 (43.8)	2812 (45.0)
**Low (< 3.1%)**	431 (18.6)	2173 (19.1)	2624 (18.9)	1260 (20.2)
**Missing**	228(9.8)	1189 (10.5)	1369 (9.9)	525 (8.4)

There are distinct trends among the area-level SDoH metrics. We used the g-test of independence to compare rates in area-level SDoH across all age groups between (1) the U09.9 cohort (reflected in Table 1, above) and (2) all SARS-CoV-2 positive patients at the same sites (shown in Additional file 1: Supplemental Table 1). Post hoc analysis showed that the U09.9 cohort had significantly lower representation in socially deprived areas than all SARS-CoV-2 positive patients. The U09.9 patient cohort had more patients in the "low" category for households with income below the poverty rate (29.6% vs. 26.3%; *p*-value < 0.01). The U09.9 cohort also had a higher percentage of patients in the "low" category for residents who are unemployed (19.2% vs. 16.1%; *p*-value < 0.01), and residents 19–64 with public health insurance (36.5% vs. 31.5%; *p*-value < 0.01). Percentages in the “high” category for residents with a college degree were similar, though statistically significant (52.5% vs. 50.7%; *p*-value < 0.01). Additionally, compared with all SARS-CoV-2 positive patients at the same sites, the U09.9 cohort skewed toward female (66.9% vs. 55.6%; *p*-value < 0.01), White (71.8% vs. 61.1%; *p*-value < 0.01), non-Hispanic individuals (79.6% vs. 74.8%; *p*-value < 0.01).

We also analyzed the uptake of the U09.9 code itself, among sites using the code. There is a rapid increase in the use of U09.9 by sites following the code’s release (Fig. 1). Usage of U09.9 post-release is compared with usage of B94.8 (“Sequelae of other specified infectious and parasitic diseases”); some sites used B94.8 at the Center for Disease Control’s (CDC) initial recommendation [32] as a placeholder code prior to U09.9’s release. Once U09.9 became available, it quickly supplanted B94.8. A visualization of the U09.9 uptake timeline by site is shown in Additional file 1: Supplemental Fig. 1.

The definition of long COVID [33] includes a wide-ranging list of symptoms and clinical features. Many of those features appear below in Fig. 2, a visualization of diagnoses that commonly co-occur with U09.9, and each other. As shown, the mix of co-occurring diagnoses as well as the clusters produced by the Louvain algorithm change when the cohort is subset into age groups. A full accounting of diagnoses co-occurring with U09.9 (i.e., within the analysis window) in at least 1% of our cohort is included as Additional file 1: Supplemental Fig. 3.

**Fig. 3:**
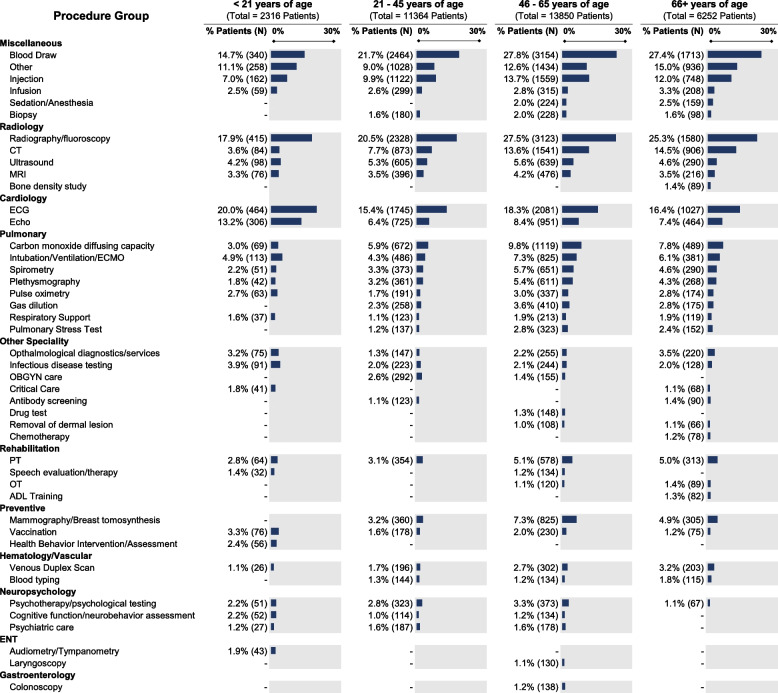
Common procedures among patients with a U09.9 code. Procedures shown occur within 60 days after a patient’s U09.9 diagnosis. Procedure records that simply reflect that an encounter took place (e.g., CPT 99212, “Office or other outpatient visit”) are excluded. Category totals represent unique patient–procedure pairs, not necessarily unique individuals. Procedure classes associated with fewer than 20 patients or less than 1.0% of the age-stratified cohort size are not shown, per the N3C download policy. Percentages in each column are shown relative to the total *n* in that column

Our findings suggest that long COVID symptoms and associated functional disability may present differently depending on the patient, but commonly fall into clusters. Conditions within a single cluster are more likely to co-occur within a single patient than conditions appearing in different clusters, allowing us to roughly subtype clinical presentations of long COVID. When stratified by age, the conditions within each cluster change somewhat, though the themes remain consistent.

N3C data also enables us to examine procedures and medications that occur in each patient’s analysis window, as shown in Fig. 3 and Additional file 1: Supplemental Fig. 3, respectively.

## Discussion

Diagnosis codes are frequently used as criteria to define patient populations. While diagnosis codes alone may not define a cohort with perfect accuracy, they are a useful mechanism to narrow a population from “everyone in the EHR” to a cohort highly enriched with the condition of interest. Our analysis of U09.9 shows that this code may serve in a similar capacity to identify long COVID patients. However, temporality and rate of uptake by providers are critical issues that must be considered. U09.9 was released for use nearly 2 years into the COVID-19 pandemic, resulting in potentially millions of patients with long COVID who “missed out” on being assigned the code. Our findings must thus be interpreted through this lens of partial and incremental adoption. More work is needed to understand clinical variability and barriers to uptake by providers.

We investigated whether the use of non-specific coding such as B94.8 (“Sequelae of other specified infectious and parasitic diseases”) could be used as a proxy for early case identification. Our findings show B94.8 use increasing among COVID patients from April 2021 to October 2021, indicating a potential shift in clinical practice patterns to code for long COVID presentation as guided by the Centers for Disease Control [32]. While B94.8 can be used for long COVID ascertainment in EHRs prior to October 2021, it should be noted that B94.8 is used to code for any sequelae of *any* infectious disease. For this reason, it may not be specific enough to rely on for highly precise long COVID case ascertainment without applying additional logic (e.g., requiring a positive COVID test prior to B94.8). Even still, it is likely the most reliable structured variable in the EHR to identify potential long COVID patients prior to October 1, 2021.

Our diagnosis clusters suggest that long COVID is not a single phenotype, but rather a collection of sub-phenotypes that may benefit from different diagnostics and treatments. Each of these clusters contains conditions and symptoms reported in existing long COVID literature [34], clearly suggests that the definition of long COVID is more expansive than lingering respiratory symptoms [35], and illustrates that long COVID can manifest differently among patients in different age groups. Notably, among the conditions represented in our clusters, six have overlap with the eight conditions identified in another recent large-scale EHR analysis as high confidence for association with PASC, suggesting the particular importance of those conditions: anosmia/dysgeusia, chronic fatigue syndrome, chest pain, palpitations, shortness of breath, and type 2 diabetes [36]. Overall, the clusters can be summarized as neurological (in blue), cardiopulmonary (in green), gastrointestinal (in purple), upper respiratory (in yellow), and comorbid conditions (in red). The clustering for the youngest patients (< 21 years of age, Fig. 2a) is the most unique, with distinct upper respiratory and gastrointestinal clusters that are not seen in other age groups. Moreover, the neurological cluster for this group also includes multiple cardiopulmonary features (e.g., dyspnea, palpitations). Patients aged 65 + (Fig. 2d) are also unique, in that they present with more chronic diseases associated with aging (e.g., congestive heart failure, atherosclerosis, atrial fibrillation) in addition to long COVID symptoms. The comorbid conditions cluster is unique in that it likely does not represent symptoms of long COVID, but rather a collection of comorbid conditions that increase in incidence as patients age. The impact of these comorbid conditions on risk and outcomes of long COVID requires further study.

Also noteworthy is the fact that the neurological cluster appears more prominently in younger groups, especially patients 21–45 years of age. Of particular note is the appearance of myalgic encephalomyelitis (listed in Systematized Nomenclature of Medicine – Clinical Terms (SNOMED CT) as “chronic fatigue syndrome,” a non-preferred term)—a disease which parallels long COVID in many ways [37–39]—in the neurological cluster across all age groups, suggesting not only frequent co-occurrence with a U09.9 diagnosis, but also co-occurrence with other neurological symptoms. The cluster differences we see among age groups make a case for age stratification when studying U09.9, and long COVID in general. Regardless, given long COVID’s heterogeneity in presentation, course, and outcome, the clustering of symptoms may prove informative for future development of classification and diagnostic criteria [40].

The common procedures around the time of U09.9 index provide insight into diagnostics and treatments currently used by providers for patients presenting with long COVID, for which treatment guidelines remain under development [41–44]. For new diseases where consensus is lacking, care is often ad hoc and informed by both the symptoms that patients present with and the available diagnostics and treatments that providers can offer. The identification and characterization of care patterns is an important step in designing future research to assess the efficacy and outcomes of these interventions. Radiographic imaging is a common occurrence across all age groups, with an average of 22.8% of patients with at least one imaging procedure in the analysis window. Electrocardiography (ECG) and echocardiography are also relatively common across all age groups, though patients younger than 21 years of age have the highest proportion (20.0% and 13.2% for ECG and echo, respectively, compared with an average of 16.7% and 7.4% across the other age groups). Pulmonary function testing shows a slight increase in frequency with more advanced age. Also of interest is the fact that some patients are receiving rehabilitation services in the 60 days after diagnosis, such as physical and occupational therapy, which lends insight into the burden of functional disability for patients with long COVID. The proportion of patients receiving rehabilitation services also rises with patient age.

Differences across age groups were less apparent in the medication analysis (Additional file 1: Supplemental Fig. 2), though the youngest patients appear slightly more likely to be prescribed medications for gastrointestinal, cardiac, and neurological indications. Unsurprisingly, respiratory system drugs were also commonly prescribed across all age groups. Interestingly, antibacterials were used frequently across all age groups; it is unclear whether patients with long COVID are more susceptible to bacterial infections, or if there may be overuse of antibiotics in the setting of fluctuating respiratory long COVID symptoms or viral infections [45, 46]. Corticosteroids were also commonly used, presumably to treat persistent inflammation as a possible mechanism mediating long COVID symptoms. The variety of medication categories seen in our analysis reflect the potential multi-system organ involvement and symptom clusters in long COVID that we see in the analysis of conditions.

We also investigated how demographics and SDoH contribute to variation in diagnosis with U09.9. When evaluating the U09.9 cohort across age groups and SDoH variables, distinct trends can be observed (see Table 1). Patients with a U09.9 diagnosis code are more likely to live in areas with low percentages of residents who are unemployed or on public health insurance. Patients living in counties with a high level of poverty make up the smallest share of the U09.9 cohort. In contrast, research shows that socially deprived areas have higher rates of COVID-19 cases and deaths [47, 48]. Given the higher rates of COVID-19, lower rates of long COVID seem unlikely. Rather, patients in deprived areas may be less likely to receive a U09.9 code in a healthcare setting, which may have downstream implications for their later identification as a long COVID patient. Moreover, a large majority of the U09.9 cohort identifies as female, White, and non-Hispanic compared to all SARS-CoV-2 positive patients at the same sites. These trends are unlikely to be an accurate reflection of the true population with long COVID, but may instead illustrate racial and social disparities in access to and experience with healthcare in the USA. Clearly, the role of access to providers and the economic means to afford long COVID care should continue to be studied for their role as contributors to disparate care and outcomes, as well as sources of research and algorithmic bias.

### Limitations

All EHR data is limited in that patients with lower access or barriers to care are less likely to be represented. Moreover, missing race and ethnicity data is likely not missing at random [49], and the inclusion of patients with missing race and/or ethnicity data in this analysis may bias interpretation of our demographic findings. EHR heterogeneity across sites may mean that a U09.9 code at one site does not quite equate to a U09.9 code at another. Moreover, we are not able to know what type of provider issued the U09.9 diagnosis (i.e., specialty), and different clinical organizations have different coding practices.

As the U09.9 code is still quite new and our sample size is limited, we cannot yet confidently label these clusters as clear “long COVID subtypes.” Rather, these clusters are intended to be hypothesis generating, with additional work underway by the RECOVER consortium to further develop and validate these clusters. It should also be noted that many symptoms are not coded in the EHR (and may, for example, be more likely to appear in free-text notes rather than diagnosis code lists). Future work will incorporate these non-structured sources of symptoms for use in our clustering methodology. The newness of the code should also be taken into account when interpreting any of our findings. The CDC has created guidance for use of the code [50]; however, despite this, as noted by an attendee at the CDC’s March 2021 Q&A session that covered U09.9, “physicians don’t speak coding” [51]. Thus, there is likely to be a disconnect between CDC’s intended use of the code and its actual application in practice, in both the billing and clinical contexts. Ioannou et al. echoed this in a recent paper, noting great variability in the documentation of long COVID across regions, medical centers, and populations [52]. We are unlikely to know the extent of this disconnect until U09.9 has been in use for a longer period of time; however, it should be assumed that some number of the patients that receive a U09.9 code may indeed be “false positives.” In future work, chart reviews of U09.9 patients will shed light on this issue.

Given the variable uptake of the U09.9 code, it is challenging to accurately identify comparator groups for this population—i.e., the absence of a U09.9 code cannot, at this time, be interpreted as the absence of long COVID. Relying solely on U09.9 to identify a complete long COVID cohort will undoubtedly miss many valid cases that are simply “unlabeled.” This will continue to be an issue in future research, especially when evaluating the effect of PASC on patient morbidity and utilization of diagnostic testing and treatments.

## Conclusions

The recent release of ICD-10-CM code U09.9 to codify long COVID will undoubtedly assist with future case ascertainment and computable phenotyping. However, a large number of patients who developed long COVID prior to October 1, 2021, continue to be burdened with symptoms, and must also be included in data-driven cohort identification efforts for trial recruitment and retrospective analyses. Considering the caveats around the rate of uptake among clinicians and late timing of the code’s release, we recommend that when characterizing long COVID using EHRs, U09.9 should not be used alone, but rather in combination with other strategies such as more complex computable phenotypes [53]. Our findings from the characterization of patients with the U09.9 diagnosis may be of use in refining phenotypes to identify pre-U09.9 patients that might have long COVID. There is a clear utility to the characterization of early use of U09.9, as it represents the first “hook” in EHR data that can be used to identify and assess current diagnostic and treatment patterns at scale. Moreover, given the heterogeneous presentation of long COVID, clustering of co-existing conditions and potential symptoms may be valuable in informing future development of more detailed criteria for the diagnosis of long COVID and its subtypes.

## Supplementary Information

**Additional file 1: Supplemental methods, supplemental table 1**, **supplemental figures 1-3.** Supplemental Methods – Description of additional methods used, including: Community detection in diagnosis analysis. network stability, age-stratified condition co-occurrence networks, and standard N3C data quality checks. **Supplemental Table 1.** – Demographic breakdown of all COVID-positive patients across 34 N3C sites. **Supplemental Figure 1.** – Uptake of U09.9 and B94.8 ICD-10_CM codes across 34 N3C sites. **Supplemental Figure 2.** – Common medications among patients with a U09.9 code. **Supplemental Figure 3.** – Common conditions among patients with a U09.9 code

## Data Availability

The N3C Data Enclave is managed under the authority of the NIH; information can be found at ncats.nih.gov/n3c/resources. Enclave data is protected, and can be accessed for COVID-related research with an approved (1) IRB protocol and (2) Data Use Request (DUR). A detailed accounting of data protections and access tiers is found in [1]. Enclave and data access instructions can be found at https://covid.cd2h.org/for-researchers; all code used to produce the analyses in this manuscript is available within the N3C Enclave to users with valid login credentials to support reproducibility.

## Abbreviations

ACS: American Community Survey
ATC: Anatomical Therapeutic Chemical
CDC: Centers for Disease Control
CPT: Current Procedural Terminology
ECG: Electrocardiography
ECMO: Extracorporeal membrane oxygenation
EHR: Electronic health record
HIPAA: Health Insurance Portability and Accountability Act
ICD-10-CM: International Classification of Diseases 10th edition, clinical modification
N3C: National COVID Cohort Collaborative
NIH: National Institutes of Health
PASC: Post-acute sequelae of SARS-CoV-2 infection
RECOVER: Researching COVID to Enhance Recovery
SDoH: Social determinants of health
SNOMED CT: Systematized Nomenclature of Medicine – Clinical Terms
WHO: World Health Organization
ZCTA: Zip Code Tabulation Area

## References

[1] Rasmussen SA, Hamosh A (2020). What’s in a name? Issues to consider when naming Mendelian disorders. Genet Med.

[2] Biesecker LG, Adam MP, Alkuraya FS, Amemiya AR, Bamshad MJ, Beck AE (2021). A dyadic approach to the delineation of diagnostic entities in clinical genomics. Am J Hum Genet.

[3] Haendel MA, McMurry JA, Relevo R, Mungall CJ, Robinson PN, Chute CG (2018). A Census of Disease Ontologies. Annual Review of Biomedical Data Science.

[4] Harrison JE, Weber S, Jakob R, Chute CG (2021). ICD-11: an international classification of diseases for the twenty-first century. BMC Med Inform Decis Mak.

[5] Piro A, Distante AE, Tagarelli A (2017). On Allusive Names for the Syphilitic Patient From the 16th to the 19th Century: The Role of Dermatopathology. Am J Dermatopathol.

[6] Coronaviridae Study Group of the International Committee on Taxonomy of Viruses. The species Severe acute respiratory syndrome-related coronavirus: classifying 2019-nCoV and naming it SARS-CoV-2. Nat Microbiol. 2020;5:536–44. 10.1038/s41564-020-0695-z PMC7095448 32123347

[7] World Health Organization. Novel Coronavirus (2019-nCoV) Situation Report - 22. 2020. https://apps.who.int/iris/bitstream/handle/10665/330991/nCoVsitrep11Feb2020-eng.pdf?sequence=1&isAllowed=y.

[8] Worobey M (2021). Dissecting the early COVID-19 cases in Wuhan. Science.

[9] Bhatt AS, McElrath EE, Claggett BL, Bhatt DL, Adler DS, Solomon SD (2021). Accuracy of ICD-10 Diagnostic Codes to Identify COVID-19 Among Hospitalized Patients. J Gen Intern Med.

[10] Bodilsen J, Leth S, Nielsen SL, Holler JG, Benfield T, Omland LH (2021). Positive Predictive Value of ICD-10 Diagnosis Codes for COVID-19. Clin Epidemiol.

[11] Lynch KE, Viernes B, Gatsby E, DuVall SL, Jones BE, Box TL (2021). Positive predictive value of COVID-19 ICD-10 diagnosis codes across calendar time and clinical setting. Clin Epidemiol.

[12] Doykov I, Hällqvist J, Gilmour KC, Mills L, Heywood WE (2020). “The long tail of Covid-19” - The detection of a prolonged inflammatory response after a SARS-CoV-2 infection in asymptomatic and mildly affected patients. F1000Res.

[13] Marshall M (2020). The lasting misery of coronavirus long-haulers. Nature.

[14] Nabavi N (2020). Long covid: How to define it and how to manage it. BMJ.

[15] Han Q, Zheng B, Daines L, Sheikh A (2022). Long-Term Sequelae of COVID-19: A Systematic Review and Meta-Analysis of One-Year Follow-Up Studies on Post-COVID Symptoms. Pathogens.

[16] Soriano JB, Murthy S, Marshall JC, Relan P, Diaz JV, WHO Clinical Case Definition Working Group on Post-COVID-19 Condition. A clinical case definition of post-COVID-19 condition by a Delphi consensus. Lancet Infect Dis. 2021. 10.1016/S1473-3099(21)00703-9. 10.1016/S1473-3099(21)00703-9 PMC8691845 34951953

[17] Nasserie T, Hittle M, Goodman SN (2021). Assessment of the Frequency and Variety of Persistent Symptoms Among Patients With COVID-19: A Systematic Review. JAMA Netw Open.

[18] McCorkell L, Assaf GS, Davis HE, Wei H, Akrami A (2021). Patient-Led Research Collaborative: embedding patients in the long COVID narrative. PAIN Reports.

[19] Davis HE, Assaf GS, McCorkell L, Wei H, Re’em Y (2021). Characterizing long COVID in an international cohort: 7 months of symptoms and their impact. EClinicalMedicine.

[20] Ledford H (2022). How common is long COVID?. Why studies give different answers Nature.

[21] RECOVER: Researching COVID to Enhance Recovery. RECOVER: Researching COVID to Enhance Recovery. https://recovercovid.org. Accessed 15 Apr 2022.

[22] Haendel MA, Chute CG, Bennett TD, Eichmann DA, Guinney J, Kibbe WA (2021). The National COVID Cohort Collaborative (N3C): Rationale, design, infrastructure, and deployment. J Am Med Inform Assoc.

[23] Pfaff ER, Girvin AT, Gabriel DL, Kostka K, Morris M, Palchuk MB (2022). Synergies between centralized and federated approaches to data quality: a report from the national COVID cohort collaborative. J Am Med Inform Assoc.

[24] Phenotype_Data_Acquisition Wiki. Github. https://github.com/National-COVID-Cohort-Collaborative/Phenotype_Data_Acquisition.

[25] US Census Bureau. American Community Survey 2015–2019 5-Year Data Release. 2021. https://www.census.gov/newsroom/press-kits/2020/acs-5-year.html.

[26] ZIP Code to ZCTA Crosswalk. https://udsmapper.org/zip-code-to-zcta-crosswalk/. Accessed 2 Nov 2022.

[27] Blondel VD, Guillaume J-L, Lambiotte R, Lefebvre E (2008). Fast unfolding of communities in large networks. J Stat Mech: Theory Exp.

[28] Pons P, Latapy M (2006). Computing Communities in Large Networks Using Random Walks. Journal of Graph Algorithms and Applications.

[29] Girvan M, Newman MEJ (2002). Community structure in social and biological networks. Proc Natl Acad Sci.

[30] WHOCC. Structure and principles. https://www.whocc.no/atc/structure_and_principles/. Accessed 11 Mar 2022.

[31] FAIR Health. Patients Diagnosed with Post-COVID Conditions [White paper]. 2022. https://s3.amazonaws.com/media2.fairhealth.org/whitepaper/asset/Patients%20Diagnosed%20with%20Post-COVID%20Conditions%20-%20A%20FAIR%20Health%20White%20Paper.pdf.

[32] CDC. Public health recommendations. Centers for Disease Control and Prevention. 2021. https://www.cdc.gov/coronavirus/2019-ncov/hcp/clinical-care/post-covid-public-health-recs.html. Accessed 5 Apr 2022.

[33] Coronavirus disease (COVID-19): Post COVID-19 condition. https://www.who.int/news-room/questions-and-answers/item/coronavirus-disease-(covid-19)-post-covid-19-condition. Accessed 28 Mar 2022.

[34] Deer RR, Rock MA, Vasilevsky N, Carmody L, Rando H, Anzalone AJ (2021). Characterizing long COVID: Deep Phenotype of a Complex Condition. EBioMedicine.

[35] Clinical Services, Systems. A clinical case definition of post COVID-19 condition by a Delphi consensus, 6 October 2021. 2021. https://www.who.int/publications/i/item/WHO-2019-nCoV-Post_COVID-19_condition-Clinical_case_definition-2021.1. Accessed 1 Apr 2022.

[36] Estiri H, Strasser ZH, Brat GA, Semenov YR, Patel CJ, Murphy SN (2021). Evolving phenotypes of non-hospitalized patients that indicate long COVID. BMC Med.

[37] Komaroff AL, Lipkin WI (2021). Insights from myalgic encephalomyelitis/chronic fatigue syndrome may help unravel the pathogenesis of postacute COVID-19 syndrome. Trends Mol Med.

[38] Proal AD, VanElzakker MB (2021). long COVID or Post-acute Sequelae of COVID-19 (PASC): An Overview of Biological Factors That May Contribute to Persistent Symptoms. Front Microbiol.

[39] Komaroff AL, Bateman L (2020). Will COVID-19 Lead to Myalgic Encephalomyelitis/Chronic Fatigue Syndrome?. Front Med.

[40] Aggarwal R, Ringold S, Khanna D, Neogi T, Johnson SR, Miller A (2015). Distinctions between diagnostic and classification criteria?. Arthritis Care Res.

[41] Mikkelsen ME, Abramoff B, Elmore JG, Kunins L. COVID-19: evaluation and management of adults following acute viral illness. UpToDate Waltham, MA: UpToDate Inc (Accessed on October 30, 2021 [Google Scholar]. 2021.

[42] Herrera JE, Niehaus WN, Whiteson J, Azola A, Baratta JM, Fleming TK (2021). Multidisciplinary collaborative consensus guidance statement on the assessment and treatment of fatigue in postacute sequelae of SARS-CoV-2 infection (PASC) patients. PM R.

[43] Fine JS, Ambrose AF, Didehbani N, Fleming TK, Glashan L, Longo M (2022). Multi-disciplinary collaborative consensus guidance statement on the assessment and treatment of cognitive symptoms in patients with post-acute sequelae of SARS-CoV-2 infection (PASC). PM and R.

[44] Maley JH, Alba GA, Barry JT, Bartels MN, Fleming TK, Oleson CV (2022). Multi-disciplinary collaborative consensus guidance statement on the assessment and treatment of breathing discomfort and respiratory sequelae in patients with post-acute sequelae of SARS-CoV-2 infection (PASC). PM and R.

[45] Harris AM, Hicks LA, Qaseem A (2016). High Value Care Task Force of the American College of Physicians and for the Centers for Disease Control and Prevention. Appropriate antibiotic use for acute respiratory tract infection in adults: Advice for high-value care from the American college of physicians and the centers for disease control and prevention. Ann Intern Med.

[46] Havers FP, Hicks LA, Chung JR, Gaglani M, Murthy K, Zimmerman RK (2018). Outpatient Antibiotic Prescribing for Acute Respiratory Infections During Influenza Seasons. JAMA Netw Open.

[47] Madlock-Brown C, Wilkens K, Weiskopf N, Cesare N, Bhattacharyya S, Riches NO (2022). Clinical, social, and policy factors in COVID-19 cases and deaths: methodological considerations for feature selection and modeling in county-level analyses. BMC Public Health.

[48] McLaughlin JM, Khan F, Pugh S, Angulo FJ, Schmitt H-J, Isturiz RE (2020). County-level Predictors of Coronavirus Disease 2019 (COVID-19) Cases and Deaths in the United States: What Happened, and Where Do We Go from Here?. Clin Infect Dis.

[49] Cook LA, Sachs J, Weiskopf NG (2021). The quality of social determinants data in the electronic health record: a systematic review. J Am Med Inform Assoc.

[50] ICD-10-CM Official Guidelines for Coding and Reporting FY 2022. https://www.cdc.gov/nchs/data/icd/10cmguidelines-FY2022-7-2022-508.pdf. Accessed 13 Oct 2022.

[51] CDC’s responses to questions or comments submitted for diagnosis code topics using the “Q & A” feature during the March 10, 2021 ICD-10 Coordination and Maintenance Committee Meeting. https://www.cdc.gov/nchs/data/icd/March-10-2021-CMQA-508.pdf. Accessed 2 Nov 2022.

[52] Ioannou GN, Baraff A, Fox A, Shahoumian T, Hickok A, O’Hare AM (2022). Rates and Factors Associated With Documentation of Diagnostic Codes for long COVID in the National Veterans Affairs Health Care System. JAMA Netw Open.

[53] Pfaff ER, Girvin AT, Bennett TD, Bhatia A, Brooks IM, Deer RR (2022). Identifying who has long COVID in the USA: a machine learning approach using N3C data. Lancet Digital Health.

