# Supplementary material for: Coding long COVID: characterizing a new disease through an ICD-10 lens
Source: BMC Med. 2023 Feb 16;21:58. doi: 10.1186/s12916-023-02737-6 (PMC9931566; doi:10.1186/s12916-023-02737-6)
Supplement: Supplementary file 1 — Additional file 1: Supplemental methods, supplemental table 1, supplemental figures 1-3. Supplemental Methods – Description of additional methods used, including: Community detection in diagnosis analysis. network stability, age-stratified condition co-occurrence networks, and standard N3C data quality checks. Supplemental Table 1. – Demographic breakdown of all COVID-positive patients across 34 N3C sites. Supplemental Figure 1. – Uptake of U09.9 and B94.8 ICD-10_CM codes across 34 N3C sites. Supplemental Figure 2. – Common medications among patients with a U09.9 code. Supplemental Figure 3. – Common conditions among patients with a U09.9 code [file 12916_2023_2737_MOESM1_ESM.pdf]

# Additional File 1

## Supplemental Methods

### Community Detection in Diagnosis Analysis

To determine conditions and symptoms that were more likely to co-occur with each other in our sample cohort than at random, we conducted a network analysis of a weighted network with nodes representing individual diagnoses, edges between nodes representing co-occurrence, and edge weights corresponding to the count of patients with both conditions. We employed three community detection algorithms: the Girvan-Newman algorithm, the Walktrap algorithm, and the Louvain algorithm. The Girvin-Newman algorithm is a hierarchical approach based on edge betweenness. Edges with high betweenness usually bridge densely connected clusters, and this algorithm detects and deletes edges with high betweenness in order to detect latent structures, assigns communities accordingly, and iteratively partitions within those communities. The Walktrap algorithm is based on the computation of a transition matrix with each element denoting the probability of one node traversing to another, and then simulating "random walks" for an n number of steps in an iterative process. Nodes are assigned to a community based on the community assignment of their neighbors within the given n distance. The Louvain algorithm iteratively uses modularity to optimize partitions, by first determining initial assignments by exchanging nodes between communities until the optimal modularity is reached, and repeating this iterative process treating the produced communities as nodes, with ties between communities. In the case of our co-occurrence network, the Louvain algorithm outperformed the Walktrap and Girvin-Newman algorithms. The Girvin-Newman algorithm may have been penalized for its choice of betweenness as a selection criterion, due to the presence of multiple edges connecting communities in this dense network. The Walktrap algorithm operates bottom-up, and is not as vulnerable to initial hierarchical partitioning that the Girvin-Newman algorithm is, but tended to categorize small communities that were connected to a central cluster via weaker links, as separate communities, effectively producing smaller, less well-separated clusters. The Louvain algorithm grouped these smaller fragmented clusters along with nearby large clusters, both improving modularity and interpretability of our final communities.

### Network Stability

We assessed the stability of the co-occurrence network using the quadratic assignment procedure (QAP). The QAP is a resampling-based method (similar to the bootstrap) that measures the correlation between two network matrices and calculates standard errors for associations. For our analysis, we use QAP to provide a statistical assessment of network similarity between the full network and a re-sampled subset of networks (75%, 50%, 25%, and 5% subsamples of patients in the original network). The resulting subsampled network correlation values against the full network were: 0.80 (5% network against the full network), 0.81 (25% network against the full network), 0.81 (50% network against the full network), and 0.81 (75% network against the full network). The results of the QAP analysis were statistically significant at an $\alpha = 0.05$, indicating high network stability.

### Age-Stratified Condition Co-occurrence Networks

For further subgroup analyses of our co-occurrence network, we presented clusters detected within age-stratified condition networks (age groups <21, 21-45, 46-55, 56-65, 65+; see **Figure 2** for results). In order to determine whether the differences in communities detected across age-stratified cohorts may be attributed to a true difference in prevalence for selected conditions across cohorts, we conducted a Pearson's chi-square test of independence. Specifically, we examined the statistical significance ($\alpha = 0.05$) of the difference across age-

groups in the prevalence for each condition that is prevalent in at least one age cohort (a total of 46 conditions). Although the observed value for the age-stratified prevalence of a few conditions was < 5, the expected value in each cell was > 5 across all conditions, allowing us to use the chi-square test instead of an exact test of independence that accounts for issues associated with small cell sizes. Of the 46 conditions present in all cohorts, the difference in prevalence was statistically significant for all 46 conditions ($p < 0.05$).

## Standard N3C Data Quality Checks

N3C's Data Ingestion and Harmonization team run a suite of quality checks against each participating site's initial data submission before those data are incorporated into the N3C secure enclave for use in research. Sites that do not pass our minimum checks are asked to remediate issues and resubmit, with that process continuing iteratively unless all checks are passed. N3C's data quality workflow is described in detail in [23]. The following list, adapted from [23], summarizes the minimum requirements for inclusion.

1. **Source common data model (CDM) conformance**. All tables required by the site's source CDM (e.g., OMOP, PCORnet, ACT, TriNetX) are present, with all required fields populated; fields that use a controlled value set (eg, "M" for male, "F" for female, etc.) are populated with valid values.
2. **Demographics.** Count of patients qualifying for COVID phenotype (e.g., COVID positive) is reasonable when compared with sites of similar size; sex, race, and ethnicity distributions are reasonable for the site's population; month of birth evenly distributed throughout the calendar year.
3. **COVID tests.** All COVID tests must be coded with a standard concept; all COVID test results must be coded with a standard concept; numbers of negative and positive COVID tests are reasonable when compared with sites of similar size.
4. **Conditions.** Clinical encounters coded with U07.1 (ICD-10 code for COVID) are present, and those encounters are distributed across various visit types (e.g., outpatient, inpatient, emergency)
5. **Encounters.** Clinical encounters are distributed across a variety of visit types (e.g., outpatient, inpatient, emergency); the distribution of visit types is reasonable when compared with similar sites; the majority of inpatient visits have valid end dates; the mean duration of visits is reasonable for that type of visit; the vast majority of visit end dates are later than or equal to the visit start date
6. **Coding completeness.** No more than 20% of records in any domain are coded with nonstandard OMOP concept IDs without further explanation (OMOP sites only); no more than 20% of records in any domain are coded with "0—No Matching Concept" without further explanation (OMOP sites only); the PERSON_ID attached to all records in domain tables must exist in the PERSON table; primary keys are valid with no duplicate rows in any table; if applied by the site, date shifting is consistent within each patient across all domains.

# Supplemental Table 1

Demographic breakdown of all COVID-positive patients across 34 N3C sites. The cohort shown in this table is composed of any COVID-positive patient at our 34 eligible sites. This cohort was used as a comparator against the U09.9-coded population in Table 1, enabling us to ascertain the ways in which the U09.9 cohort differs from the COVID-positive cohort from the same sites.

| | Age <21 | 21-45 | 46-65 | 66+ |
|---|---|---|---|---|
| | n = 470628 | n = 795691 | n = 578108 | n = 310304 |
| **Person-level variables** | | | | |
| **Sex (%)** | | | | |
| **female** | 238536 (50.7) | 476989 (59.9) | 317034 (54.8) | 167230 (53.9) |
| **male** | 231929 (49.3) | 318156 (40.0) | 260910 (45.1) | 142999 (46.1) |

| | | | | |
|---|---|---|---|---|
| unknown | 163 (0.0) | 546 (0.1) | 164 (0.0) | 75 (0.0) |
| **Race (%)** | | | | |
| American Indian or Alaska Native | 3821 (0.8) | 7258 (0.9) | 5206 (0.9) | 2022 (0.7) |
| Asian | 13318 (2.8) | 22631 (2.8) | 13982 (2.4) | 6959 (2.2) |
| Black | 95704 (20.3) | 153981 (19.4) | 102186 (17.7) | 41925 (13.5) |
| Hawaiian/Pac Isldr. | 1181 (0.3) | 2423 (0.3) | 1380 (0.2) | 1181 (0.3) |
| White | 247448 (52.6) | 461896 (58.0) | 376793 (65.2) | 230635 (74.3) |
| Other | 16430 (3.5) | 3404 (0.4) | 1192 (0.2) | 433 (0.1) |
| Unknown | 92726 (19.7) | 144098 (18.1) | 77369 (13.4) | 27896 (9.0) |
| **Ethnicity (%)** | | | | |
| Hispanic/Latino | 99803 (21.2) | 137042 (17.2) | 75073 (13.0) | 21137 (6.8) |
| Not Hispanic/Latino | 316257 (67.2) | 576019 (72.4) | 452401 (78.3) | 268131 (86.4) |
| Unknown | 54566 (11.6) | 82628 (10.4) | 50633 (8.8) | 21033 (6.8) |
| **Area-level social determinants of health (ZIP-code level)** | | | | |
| **Households with Income below poverty (%)** | | | | |
| High (>15.30%) | 135422 (28.8) | 249764 (31.4) | 176233 (30.5) | 95258 (30.7) |
| Medium (7.92-15.30%) | 146855 (31.2) | 252268 (31.7) | 180741 (31.3) | 102731 (33.1) |
| Low (<7.92%) | 128366 (27.3) | 197010 (24.8) | 155636 (26.9) | 85885 (27.7) |
| Missing | 59985 (12.7) | 96649 (12.1) | 65498 (11.3) | 26430 (8.5) |
| **Residents with college degree (%)** | | | | |
| High (>17.54%) | 240982 (51.2) | 408665 (51.4) | 284551 (49.2) | 158815 (51.2) |
| Medium (10.27-17.54%) | 119727 (25.4) | 203280 (25.5) | 155001 (26.8) | 84681 (27.3) |
| Low (<10.27%) | 50138 (10.7) | 87267 (11.0) | 73138 (12.7) | 40422 (13.0) |
| Missing | 59781 (12.7) | 96479 (12.1) | 65418 (11.3) | 26386 (8.5) |
| **Residents 19-64 with public health insurance (%)** | | | | |
| High (>22.50%) | 101181 (21.5) | 178539 (22.4) | 133596 (23.1) | 71812 (23.1) |
| Medium (12.99-22.50%) | 158161 (33.6) | 271128 (34.1) | 199770 (34.6) | 112090 (36.1) |
| Low (<12.99%) | 151510 (32.2) | 249492 (31.4) | 179269 (31.0) | 99973 (32.2) |
| Missing | 59776 (12.7) | 96532 (12.1) | 65473 (11.3) | 26429 (8.5) |
| **Residents 19-64 Unemployed (%)** | | | | |
| High (>5.7%) | 125466 (26.7) | 216035 (27.2) | 164593 (28.5) | 89937 (29.0) |
| Medium (3.1%-5.7%) | 213750 (45.4) | 355978 (44.7) | 252948 (43.8) | 139891 (45.1) |
| Low (<3.1%) | 71634 (15.2) | 127142 (16.0) | 95092 (16.4) | 54040 (17.4) |
| Missing | 59778 (12.7) | 96536 (12.1) | 65475 (11.3) | 26436 (8.5) |

# Supplemental Figure 1

**Uptake of U09.9 and B94.8 across 34 N3C sites.** Each box represents one of our 34 sites, with counts of U09.9 and B94.8 diagnosis codes plotted over time. Most sites follow a similar uptake pattern, with U09.9 use (blue) rising since its release on October 1, 2021, and B94.8 (pink) decreasing or plateauing after that date. Note that because N3C sites refresh their data on different cycles, some sites have more recent data than others, leading to different end dates across these plots.

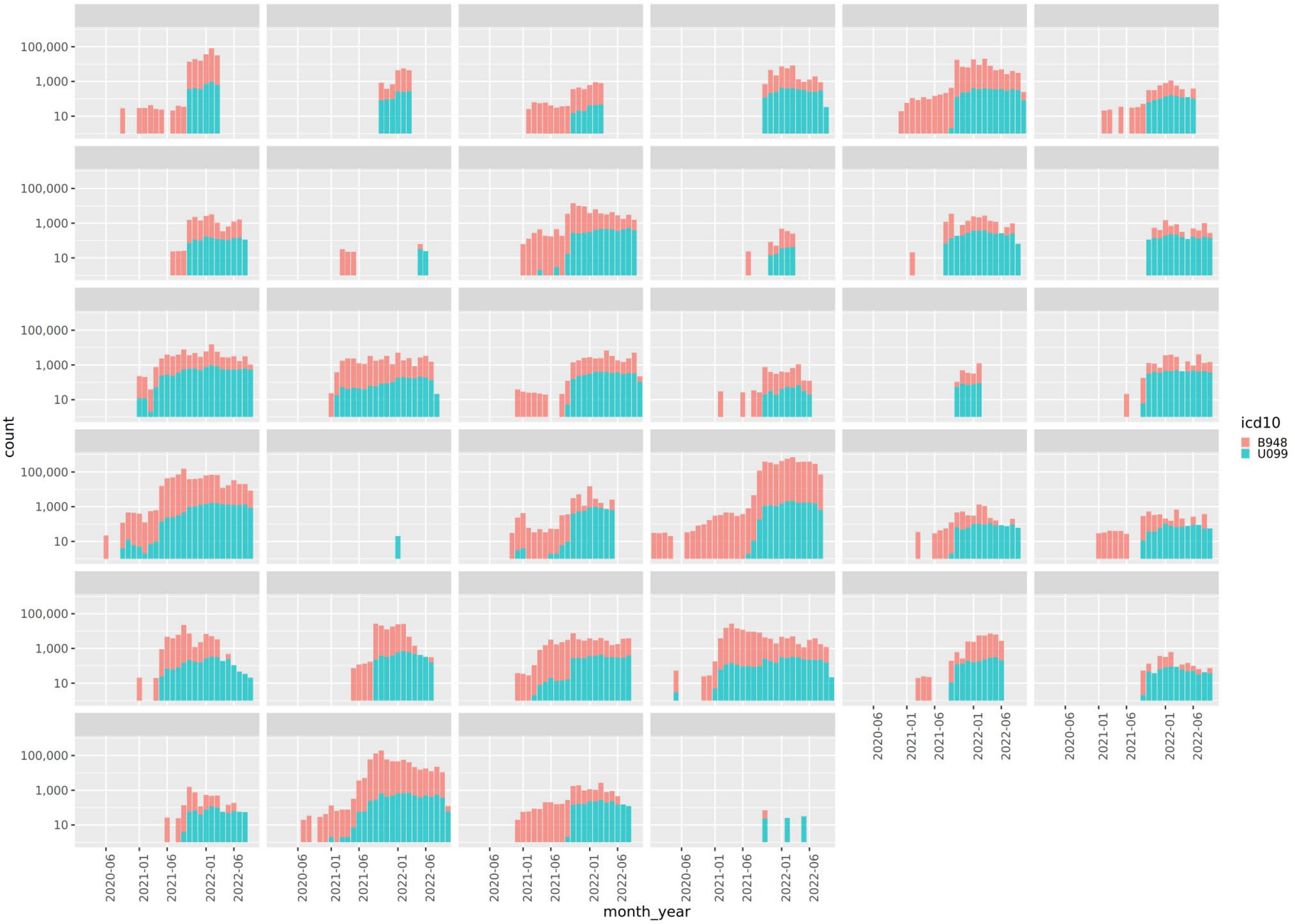


# Supplemental Figure 2

**Common medications among patients with a U09.9 code.** Medications shown occur within 60 days after a patient's U09.9 diagnosis, and do *not* occur prior to the U09.9 (i.e., new medications). Medications are coded using the ATC terminology. Because a single drug can have multiple ATC codes, some medications are counted in more than one category. Category totals represent unique patient - drug pairs, not necessarily unique individuals. Medication classes associated with fewer than 20 patients or less than 0.5% of the age-stratified cohort size are not shown, per N3C download policy. Percentages in each column are shown relative to the total *n* in that column.

When using EHR data, it can be difficult to discern indication from drug records, particularly when drugs are recorded at the ingredient level, and particularly when those ingredients can be used in a wide variety of medications and medication forms. For this reason, the categories of Opthalmologicals; Otologicals;

Corticosteroids, Dermatological Preparations; and Blood Substitutes and Perfusion Solutions are artificially inflated. They are included for completeness, but should not be interpreted at face value.

| Medication Group | < 21 years of age (Total = 2316 Patients) | 21 - 45 years of age (Total = 11364 Patients) | 46 - 65 years of age (Total = 13850 Patients) | 66+ years of age (Total = 6252 Patients) |
|---|---|---|---|---|
| | % Patients (N) 0% 7% | % Patients (N) 0% 7% | % Patients (N) 0% 7% | % Patients (N) 0% 7% |
| **Sensory Organ Drugs** | | | | |
| Ophthalmologicals | 6.6% (152) | 4.3% (487) | 5.3% (601) | 3.9% (246) |
| Otologicals | 4.8% (111) | 2.6% (300) | 3.3% (375) | 2.1% (129) |
| Ophthalmological And Otological Preparations | 3.5% (82) | 1.9% (212) | 2.2% (248) | 1.4% (88) |
| **Alimentary Tract and Metabolism Drugs** | | | | |
| Antidiarrheals, Intestinal Antiinflammatory/Antiinfective Agents | 3.8% (88) | 3.6% (404) | 4.1% (469) | 3.2% (200) |
| Drugs For Acid Related Disorders | 3.2% (74) | 1.3% (145) | 1.8% (200) | 1.3% (83) |
| Mineral Supplements | 2.8% (64) | 1.1% (122) | 1.7% (195) | 1.4% (89) |
| Vitamins | 1.6% (36) | 1.7% (196) | 2.0% (227) | 1.3% (81) |
| Stomatological Preparations | 1.9% (44) | - | 1.3% (152) | - |
| Drugs For Constipation | 1.3% (31) | - | 1.1% (124) | - |
| Antiemetics And Antinauseants | 1.6% (38) | - | - | - |
| **Respiratory System Drugs** | | | | |
| Drugs For Obstructive Airway Diseases | 4.9% (113) | 4.5% (516) | 4.8% (544) | 3.4% (215) |
| Nasal Preparations | 3.3% (77) | 2.6% (293) | 3.0% (344) | 2% (126) |
| Cough And Cold Preparations | 1.2% (28) | 1.2% (134) | 1.5% (170) | 1.1% (70) |
| Antihistamines For Systemic Use | 2.0% (47) | - | 1.1% (121) | - |
| Throat Preparations | 2.0% (47) | - | - | - |
| **Cardiovascular System Drugs** | | | | |
| Cardiac Therapy | 3.4% (78) | 1.5% (173) | 2.4% (269) | 2.1% (134) |
| Vasoprotectives | 3.2% (74) | 1.4% (164) | 1.9% (217) | 1.6% (99) |
| Diuretics | 1.2% (27) | - | 1.2% (139) | 1.1% (67) |
| Beta Blocking Agents | - | - | 1.5% (165) | - |
| Lipid Modifying Agents | - | - | 1.7% (189) | 1.1% (66) |
| Agents Acting On The Renin-Angiotensin System | - | - | 1.2% (133) | 1.1% (70) |
| **Nervous System Drugs** | | | | |
| Analgesics | 3.2% (73) | 1.5% (166) | 1.9% (214) | 1.2% (73) |
| Anesthetics | 2.5% (57) | 1.4% (156) | 1.6% (184) | 1.3% (82) |
| Psycholeptics | 1.7% (39) | 1.3% (148) | 1.7% (193) | 1.3% (84) |
| Psychoanaleptics | 1.7% (40) | 1.2% (136) | 1.2% (137) | - |
| **Blood and Blood Forming Organ Drugs** | | | | |
| Blood Substitutes And Perfusion Solutions | 4.1% (95) | 2.9% (326) | 3.7% (424) | 2.7% (171) |
| Antithrombotic Agents | 2.2% (50) | - | 1.4% (155) | 1.4% (86) |
| Antianemic Preparations | - | 1.1% (130) | 1.1% (122) | - |
| **Various Drug Classes in ATC** | | | | |
| Contrast Media | 2.8% (66) | 3.6% (414) | 4.0% (452) | 4.0% (249) |
| Diagnostic Agents | 1.0% (24) | 1.0% (117) | 1.0% (118) | 1.1% (67) |
| **Dermatologicals** | | | | |
| Corticosteroids, Dermatological Preparations | 3.2% (73) | 2.4% (273) | 2.8% (315) | 1.5% (96) |
| Antipruritics, Incl. Antihistamines, Anesthetics, Etc. | 2.0% (47) | - | 1.1% (120) | - |
| **Antiinfectives for Systemic Use** | | | | |
| Antibacterials For Systemic Use | 3.1% (71) | 1.9% (220) | 2.7% (311) | 2.2% (140) |
| Vaccines | 1.6% (36) | - | - | - |
| **Genitourinary System and Sex Hormones** | | | | |
| Gynecological Antiinfectives And Antiseptics | 1.6% (37) | 1.1% (125) | 1.4% (158) | - |
| Other Gynecologicals | 2.2% (51) | - | - | - |
| **Musculo-Skeletal System Drugs** | | | | |
| Antiinflammatory And Antirheumatic Products | 1.9% (45) | 1.2% (140) | 1.8% (201) | - |
| Topical Products For Joint And Muscular Pain | 1.5% (35) | - | - | - |
| **Other Systemic Hormonal Preparations** | | | | |
| Corticosteroids For Systemic Use | 1.9% (45) | 1.6% (177) | 1.8% (210) | 1.3% (80) |

# Supplemental Figure 3

**Common conditions among patients with a U09.9 code.** Conditions shown occur within 60 days after a patient's U09.9 diagnosis. Conditions associated with fewer than 20 patients or less than 1% of the age-stratified cohort size are not shown.

| Condition | < 21 years of age (Total = 2316 Patients) % Patients (N) | 21 - 45 years of age (Total = 11364 Patients) % Patients (N) | 46 - 65 years of age (Total = 13850 Patients) % Patients (N) | 66+ years of age (Total = 6252 Patients) % Patients (N) |
|---|---|---|---|---|
| Dyspnea | 15.4% (357) | 30.6% (3481) | 34.2% (4737) | 31.9% (1994) |
| Essential hypertension | 1.1% (25) | 10.2% (1159) | 26.8% (3706) | 38.9% (2434) |
| Fatigue | 15.2% (352) | 18.0% (2040) | 17.8% (2463) | 15.0% (938) |
| Cough | 12.7% (295) | 13.8% (1564) | 14.3% (1985) | 14.4% (900) |
| Chest pain | 10.9% (253) | 13.9% (1577) | 11.1% (1533) | 7.0% (439) |
| Anxiety disorder | 7.3% (169) | 14.5% (1652) | 11.2% (1553) | 7.7% (483) |
| Hyperlipidemia | - | 3.6% (409) | 10.9% (1506) | 19.9% (1244) |
| esophagitis | 2.7% (62) | 6.9% (785) | 10.1% (1399) | 13.3% (833) |
| Chronic cough | 7.9% (182) | 8.0% (913) | 8.7% (1204) | 9.1% (570) |
| Chronic pain | 4.9% (113) | 8.1% (920) | 9.8% (1360) | 9.0% (564) |
| Chronic fatigue syndrome | 3.9% (90) | 7.9% (893) | 8.3% (1145) | 8.3% (518) |
| Type 2 diabetes mellitus w/o complication | - | 3.4% (382) | 10.3% (1432) | 13.7% (854) |
| Headache | 8.6% (200) | 7.8% (889) | 6.7% (931) | 4.4% (272) |
| Finding related to attentiveness | 3.5% (81) | 9.1% (1039) | 9.4% (1302) | 5.4% (340) |
| Obstructive sleep apnea syndrome | 1.1% (26) | 4.6% (518) | 9.7% (1349) | 10.7% (671) |
| Dizziness and giddiness | 5.6% (130) | 6.7% (765) | 6.3% (872) | 5.8% (362) |
| Depressive disorder | 2.7% (63) | 7.4% (843) | 6.9% (952) | 5.8% (365) |
| Palpitations | 4.2% (97) | 8.8% (999) | 5.9% (823) | 3.2% (199) |
| Obesity | 2.3% (54) | 5.9% (674) | 6.8% (947) | 5.9% (366) |
| Tachycardia | 5.4% (125) | 7.2% (819) | 4.4% (613) | 2.6% (162) |
| Uncomplicated asthma | 3.8% (88) | 6.5% (741) | 5.7% (784) | 4.1% (254) |
| Vitamin D deficiency | 1.7% (40) | 5.0% (568) | 5.8% (802) | 5.4% (339) |
| Abnormal findings on diagnostic imaging of lung | 1.1% (26) | 2.5% (287) | 5.2% (718) | 7.8% (487) |
| Hypothyroidism | - | 2.6% (301) | 5.4% (754) | 8.8% (549) |
| Type 2 diabetes mellitus | - | 1.8% (204) | 6.0% (832) | 8.7% (544) |
| Morbid obesity | - | 6.0% (683) | 6.5% (905) | 3.9% (245) |
| angina pectoris | - | - | 3.5% (488) | 11.5% (718) |
| Mixed hyperlipidemia | - | 1.6% (182) | 5.5% (765) | 8.6% (535) |
| Generalized anxiety disorder | 2.7% (62) | 5.8% (663) | 3.9% (544) | 2.7% (166) |
| Malaise | 1.9% (45) | 3.2% (361) | 4.2% (585) | 5.5% (345) |
| Fever | 7.9% (183) | 1.9% (212) | 1.7% (242) | 1.5% (95) |
| Blood chemistry abnormal | 1.6% (36) | 3.4% (388) | 3.6% (505) | 4.2% (265) |
| Abdominal pain | 6.0% (140) | 2.9% (327) | 2.2% (308) | 2.4% (147) |
| Sensory disorder of smell and/or taste | 3.6% (84) | 4.1% (462) | 3.4% (468) | 2.6% (164) |
| Hypoxemia | - | 1.8% (206) | 3.9% (538) | 6.0% (376) |
| Anemia | 1.7% (39) | 2.1% (240) | 2.6% (365) | 5.6% (349) |
| Muscle pain | 2.8% (64) | 4.1% (461) | 3.7% (518) | 2.3% (146) |
| Nausea | 4.1% (95) | 3.4% (390) | 2.8% (386) | 2.3% (142) |
| Joint pain | 2.6% (60) | 3.5% (403) | 3.9% (543) | 2.3% (142) |
| Chronic obstructive lung disease | - | - | 3.5% (481) | 8.2% (510) |
| Diarrhea | 2.9% (68) | 2.9% (328) | 2.6% (362) | 2.9% (180) |
| Sexually active | - | 5.3% (606) | 3.5% (490) | 2.7% (170) |
| Electrocardiogram abnormal | 2.1% (48) | 2.2% (253) | 2.9% (397) | 4.1% (259) |
| Insomnia | - | 3.2% (366) | 3.9% (538) | 4.0% (249) |
| Allergic rhinitis | 3.3% (76) | 2.8% (314) | 2.8% (391) | 2.2% (140) |
| Constipation | 3.3% (76) | 1.8% (203) | 1.9% (260) | 2.8% (177) |
| Acute pharyngitis | 5.0% (116) | 2.6% (299) | 1.7% (230) | 1.2% (76) |
| Pneumonia | - | 1.4% (154) | 2.4% (332) | 4.6% (286) |
| Asthenia | - | 1.9% (216) | 2.8% (381) | 5.2% (328) |
| Pneumonia caused by SARS-CoV-2 | - | 1.8% (201) | 3.5% (489) | 4.6% (287) |
| Low back pain | - | 2.6% (292) | 3.4% (468) | 3.9% (242) |
| Nasal congestion | 3.2% (73) | 2.6% (296) | 2.2% (306) | 1.8% (115) |
| Acute upper respiratory infection | 3.9% (90) | 2.5% (279) | 1.7% (240) | 1.6% (103) |
| Congestive heart failure | - | - | 1.9% (264) | 6.5% (404) |
| Pure hypercholesterolemia | - | 1.0% (119) | 3.0% (412) | 4.9% (308) |
| Neck pain | - | 2.5% (287) | 3.0% (414) | 2.4% (147) |
| Sexually active with men | - | 3.7% (421) | 2.7% (375) | 2.7% (167) |
| Hyperglycemia due to type 2 diabetes mellitus | - | 1.2% (138) | 3.6% (498) | 3.8% (236) |
| Disorder of body system | 1.6% (38) | 1.7% (198) | 1.9% (270) | 2.7% (170) |
| Nicotine dependence | - | 3.0% (336) | 3.5% (479) | 2.1% (134) |
| Disorder of lung | - | 1.4% (158) | 3.1% (429) | 4.1% (256) |
| Prediabetes | - | 1.7% (191) | 3.5% (488) | 3.6% (228) |
| Viral disease | 3.8% (88) | 1.3% (149) | 1.4% (191) | 1.4% (85) |
| Cardiac arrhythmia | 3.1% (71) | 2.5% (284) | 1.2% (170) | 1.6% (98) |
| Postoperative state | 1.1% (25) | 1.4% (159) | 2.2% (309) | 3.5% (218) |
| Iron deficiency anemia | 1.1% (25) | 2.2% (252) | 2.0% (275) | 2.5% (156) |
| Eruption | 2.6% (60) | 1.7% (192) | 1.6% (216) | 1.4% (87) |
| Mild intermittent asthma | 2.2% (51) | 2.5% (287) | 2.0% (274) | 1.3% (83) |
| Uncomplicated moderate persistent asthma | 2.3% (53) | 2.2% (249) | 2.0% (275) | 1.6% (97) |
| Paresthesia | 1.1% (25) | 2.9% (326) | 2.5% (347) | 1.5% (92) |
| Nausea and vomiting | 2.9% (68) | 1.8% (210) | 1.3% (179) | 1.2% (77) |
| Dependence on supplemental oxygen | - | - | 3.0% (412) | 4.3% (270) |
| Atrial fibrillation | - | - | 1.3% (177) | 5.8% (362) |
| Abnormal breathing | 1.8% (41) | 1.3% (143) | 2.1% (286) | 1.9% (119) |
| Loss of consciousness | 2.5% (57) | 1.6% (178) | 1.3% (178) | 1.6% (97) |
| Wheezing | 2.3% (54) | 1.8% (202) | 1.6% (218) | 1.4% (85) |
| Acute hypoxemic respiratory failure | - | - | 2.0% (277) | 3.1% (195) |
| Shoulder joint pain | - | 1.3% (148) | 2.9% (399) | 2.7% (168) |
| Currently not sexually active | - | 1.3% (149) | 1.9% (257) | 3.7% (231) |
| Paroxysmal atrial fibrillation | - | - | 1.2% (160) | 5.1% (320) |
| Localized edema | - | 1.0% (114) | 2.0% (282) | 3.7% (229) |
| Acute renal failure syndrome | - | - | 1.3% (174) | 3.2% (198) |
| Sleep disorder | 1.9% (43) | 1.8% (209) | 1.8% (244) | 1.2% (78) |
| Hypokalemia | - | - | 1.7% (229) | 2.3% (142) |

| | | | | |
|---|---|---|---|---|
| Backache | - | 1.7% (196) | 2.3% (322) | 2.2% (136) |
| Fibromyalgia | - | 1.8% (202) | 2.7% (370) | 2.0% (124) |
| Disorder of nasal cavity | 1.7% (40) | 1.7% (188) | 1.5% (211) | 1.5% (94) |
| Migraine | 1.6% (38) | 2.7% (305) | 2.0% (279) | - |
| Vitamin B deficiency | - | 1.6% (185) | 2.1% (290) | 2.7% (166) |
| Nervous system symptoms | 1.3% (30) | 1.7% (195) | 1.8% (244) | 1.6% (102) |
| Amnesia | - | 1.5% (170) | 2.3% (314) | 2.5% (159) |
| Cardiomegaly | 1.0% (24) | - | 1.5% (206) | 2.6% (160) |
| Osteoarthritis of knee | - | - | 2.5% (351) | 3.6% (224) |
| Interstitial lung disease | - | - | 2.3% (318) | 3.6% (224) |
| Osteoarthritis | - | - | 2.0% (271) | 3.9% (245) |
| Polyneuropathy | - | 1.2% (142) | 2.0% (274) | 2.6% (160) |
| General finding of observation of patient | 1.9% (45) | 1.5% (169) | 1.4% (188) | 1.2% (76) |
| Pulmonary embolism | - | 1.1% (122) | 2.0% (283) | 2.8% (172) |
| Chronic kidney disease due to hypertension | - | - | 1.2% (163) | 4.4% (278) |
| Chronic hypoxemic respiratory failure | - | - | 2.2% (305) | 3.4% (210) |
| Moderate recurrent major depression | - | 2.1% (240) | 2.0% (278) | 1.7% (104) |
| Urinary tract infectious disease | - | - | 1.2% (167) | 3.1% (196) |
| Chronic kidney disease due to type 2 diabetes mellitus | - | - | 1.2% (170) | 3.8% (237) |
| Migraine w/o aura | 1.8% (42) | 2.3% (257) | 1.5% (209) | - |
| Heart failure | - | - | 1.3% (184) | 3.6% (227) |
| Acquired absence of organ | - | - | 1.5% (210) | 2.5% (155) |
| Reduced mobility | - | 1.2% (141) | 1.8% (244) | 2.5% (154) |
| Dysphagia | - | 1.1% (120) | 1.6% (228) | 2.3% (146) |
| Sleep apnea | - | 1.2% (140) | 2.0% (272) | 1.8% (113) |
| Dysuria | - | 1.5% (172) | 1.6% (219) | 2.0% (125) |
| Pain of right knee joint | - | 1.3% (152) | 1.9% (258) | 2.0% (124) |
| Disorder following viral disease | 5.2% (120) | - | - | - |
| Chronic sinusitis | - | 1.7% (191) | 1.8% (251) | 1.6% (97) |
| Disorder of bone | - | - | 1.2% (166) | 3.8% (237) |
| Dependence on enabling machine or device | - | - | 2.0% (282) | 2.7% (171) |
| Postviral fatigue syndrome | 1.0% (24) | 1.3% (152) | 1.4% (199) | 1.0% (65) |
| Hypertensive heart failure | - | - | 1.0% (141) | 3.3% (204) |
| Solitary nodule of lung | - | - | 1.7% (235) | 2.9% (183) |
| Seasonal allergic rhinitis | 1.3% (31) | 1.1% (127) | 1.2% (173) | 1.1% (66) |
| Major depression, single episode | - | 1.5% (167) | 1.6% (222) | 1.6% (97) |
| Multisystem inflammatory syndrome | 2.8% (66) | - | - | - |
| Fibrosis of lung | - | - | 1.4% (199) | 2.7% (168) |
| Chronic kidney disease | - | - | 1.1% (154) | 3.1% (193) |
| Hypo-osmolality and or hyponatremia | - | - | - | 2.4% (149) |
| Disorder of soft tissue | - | 1.0% (116) | 1.6% (215) | 1.7% (104) |
| Bradycardia | 1.2% (28) | - | - | 1.9% (120) |
| Osteoporosis | - | - | - | 4.4% (274) |
| Dehydration | 2.1% (49) | - | - | 1.3% (79) |
| Chronic kidney disease stage 3 | - | - | - | 4.0% (252) |
| Chronic pain syndrome | - | 1.0% (116) | 1.6% (224) | 1.6% (103) |
| Complication due to diabetes mellitus | - | - | 1.8% (246) | 2.2% (139) |
| Lumbago with sciatica | - | 1.1% (125) | 1.4% (196) | 1.5% (96) |
| Posttraumatic stress disorder | - | 2.2% (249) | 1.8% (248) | - |
| Bronchitis | - | 1.4% (158) | 1.3% (185) | 1.3% (81) |
| Musculoskeletal finding | - | 1.1% (121) | 1.4% (192) | 1.6% (97) |
| Epigastric pain | - | 1.6% (184) | 1.3% (181) | - |
| Spasm | - | 1.2% (141) | 1.4% (199) | 1.3% (80) |
| Pain | - | 1.4% (164) | 1.5% (206) | 1.1% (66) |
| Polyneuropathy due to type 2 diabetes mellitus | - | - | 1.3% (185) | 2.4% (152) |
| Anesthesia of skin | - | 2.1% (237) | 1.7% (238) | - |
| Hip pain | - | - | 1.6% (227) | 2.1% (133) |
| Pulmonary emphysema | - | - | - | 2.6% (160) |
| Uncomplicated mild persistent asthma | 2.3% (54) | 1.3% (149) | - | - |
| Chronic diastolic heart failure | - | - | - | 3.2% (203) |
| Visual disturbance | - | 1.2% (132) | 1.3% (181) | - |
| Pain in right lower limb | - | - | 1.2% (166) | 1.2% (72) |
| Pain of left knee region | - | 1.0% (114) | 1.8% (250) | 1.7% (104) |
| Snoring | - | 1.6% (185) | 1.7% (242) | - |
| Immunodeficiency disorder | - | - | 1.3% (183) | 1.8% (110) |
| Chronic rhinitis | - | 1.0% (117) | 1.1% (149) | 1.1% (71) |
| Low blood pressure | 1.0% (24) | - | - | 1.4% (88) |
| Acquired hypothyroidism | - | - | 1.1% (158) | 2.0% (122) |
| Vomiting | 2.9% (67) | - | - | - |
| Radiology result abnormal | - | - | 1.3% (179) | 1.7% (109) |
| Atelectasis | - | - | - | 1.7% (108) |
| Edema | - | - | 1.1% (155) | 1.9% (120) |
| Attention deficit hyperactivity disorder | 1.5% (35) | 1.4% (156) | - | - |
| Disorder of nervous system due to type 2 diabetes mellitus | - | - | 1.1% (155) | 1.8% (114) |
| Finding of frequency of urination | - | - | 1.1% (154) | 1.9% (121) |
| Hyperglycemia | - | - | 1.3% (176) | 1.6% (97) |
| Ventricular premature complex | - | - | 1.0% (139) | 1.8% (110) |
| Lumbar radiculopathy | - | - | 1.4% (196) | 1.5% (95) |
| Thrombocytopenic disorder | - | - | - | 1.6% (101) |
| Non-scarring alopecia | - | 1.4% (164) | 1.5% (202) | - |
| Sexually active with women | - | 1.3% (153) | - | 1.6% (98) |
| Chronic kidney disease stage 3A | - | - | - | 2.7% (171) |
| Steatosis of liver | - | 1.1% (127) | 1.6% (226) | - |

| | | | | |
|---|---|---|---|---|
| Benign prostatic hyperplasia | - | - | - | 2.4% (151) |
| Rheumatoid arthritis | - | - | 1.1% (150) | 1.5% (93) |
| Degeneration of lumbar intervertebral disc | - | - | 1.2% (163) | 1.4% (90) |
| Loss of sense of smell | - | 1.4% (161) | 1.2% (165) | - |
| Lumbar spondylosis | - | - | 1.2% (165) | 1.4% (87) |
| Pulmonary function studies abnormal | - | - | 1.3% (174) | 1.2% (77) |
| Hypertensive heart and renal disease with (congestive) heart failure | - | - | - | 2.0% (128) |
| Dysphonia | - | - | 1.3% (182) | 1.1% (66) |
| Benign prostatic hyperplasia with outflow obstruction | - | - | - | 2.3% (143) |
| Generalized abdominal pain | 2.3% (53) | - | - | - |
| Loss of appetite | 1.1% (26) | - | - | 1.2% (73) |
| Muscle weakness | - | - | 1.2% (160) | 1.1% (67) |
| Pain in left lower limb | - | - | 1.1% (153) | 1.1% (69) |
| Coronary artery graft present | - | - | - | 2.0% (124) |
| Chronic congestive heart failure | - | - | - | 2.1% (133) |
| Pulmonary hypertension | - | - | - | 2.1% (131) |
| Old myocardial infarction | - | - | - | 2.0% (123) |
| Hypersomnia | - | 1.1% (128) | 1.1% (154) | - |
| Bacterial infectious disease | - | 1.2% (133) | - | - |
| Chronic systolic heart failure | - | - | - | 2.1% (129) |
| Pleural effusion | - | - | - | 1.9% (120) |
| Posterior rhinorrhea | - | 1.0% (119) | - | 1.1% (67) |
| Acute sinusitis | 1.0% (24) | 1.1% (124) | - | - |
| Chronic kidney disease stage 3B | - | - | - | 2.0% (126) |
| Taste sense altered | - | 1.0% (119) | 1.0% (144) | - |
| Pain in left foot | - | - | 1.0% (144) | 1.0% (63) |
| Peripheral vascular disease | - | - | - | 1.9% (116) |
| Atherosclerosis of aorta | - | - | - | 1.7% (108) |
| Bronchiectasis | - | - | - | 1.8% (110) |
| Aortocoronary bypass graft present | - | - | - | 1.6% (98) |
| Inflammatory disorder | 1.8% (42) | - | - | - |
| Anemia in chronic kidney disease | - | - | - | 1.6% (100) |
| Systemic disease | 1.8% (41) | - | - | - |
| Acute exacerbation of chronic obstructive airways disease | - | - | - | 1.5% (94) |
| Menopause present | - | - | - | 1.7% (109) |
| Sensorineural hearing loss, bilateral | - | - | - | 1.7% (105) |
| Diaphragmatic hernia | - | - | - | 1.6% (98) |
| Gout | - | - | - | 1.6% (101) |
| Diastolic heart failure | - | - | - | 1.5% (95) |
| Panic disorder w/o agoraphobia | - | 1.6% (181) | - | - |
| Peripheral circulatory disorder due to type 2 diabetes mellitus | - | - | - | 1.5% (93) |
| Inflammatory dermatosis | 1.5% (34) | - | - | - |
| Spinal stenosis of lumbar region | - | - | - | 1.6% (98) |
| Tear film insufficiency | - | - | - | 1.6% (100) |
| Primary malignant neoplasm of prostate | - | - | - | 1.5% (96) |
| Supraventricular tachycardia | - | - | - | 1.5% (91) |
| Nuclear senile cataract | - | - | - | 1.5% (92) |
| Restless legs | - | - | - | 1.4% (87) |
| Hypomagnesemia | - | - | - | 1.3% (83) |
| Migraine with aura | - | 1.5% (166) | - | - |
| Pain in pelvis | - | 1.5% (168) | - | - |
| Chronic kidney disease stage 2 | - | - | - | 1.5% (91) |
| Systolic heart failure | - | - | - | 1.3% (81) |
| Chronic kidney disease stage 4 | - | - | - | 1.3% (80) |
| Centriacinar emphysema | - | - | - | 1.4% (85) |
| Hyperkalemia | - | - | - | 1.2% (74) |
| Non-rheumatic mitral valve stenosis with regurgitation | - | - | - | 1.3% (81) |
| Impacted cerumen | - | - | - | 1.4% (85) |
| Finding related to pregnancy | - | 1.4% (155) | - | - |
| Disorder of kidney and/or ureter | - | - | - | 1.3% (80) |
| Hearing loss | - | - | - | 1.2% (74) |
| Leukocytosis | - | - | - | 1.2% (75) |
| Angina co-occurrent and due to coronary arteriosclerosis | - | - | - | 1.2% (77) |
| Cardiomyopathy | - | - | - | 1.3% (81) |
| Heart disease | - | - | - | 1.3% (79) |
| Noninflammatory disorder of the vagina | - | 1.3% (147) | - | - |
| Localized, primary osteoarthritis of the shoulder region | - | - | - | 1.3% (80) |
| Osteoarthritis of hip | - | - | - | 1.2% (76) |
| Familial dysautonomia | 1.3% (29) | - | - | - |
| Actinic keratosis | - | - | - | 1.3% (79) |
| Localized enlarged lymph nodes | - | - | - | 1.2% (76) |
| Senile hyperkeratosis | - | - | - | 1.2% (78) |
| Otitis media | 1.2% (27) | - | - | - |
| Altered mental status | - | - | - | 1.0% (65) |
| Right bundle branch block | - | - | - | 1.0% (64) |
| Carotid artery obstruction | - | - | - | 1.1% (69) |
| Persistent atrial fibrillation | - | - | - | 1.1% (71) |
| Retention of urine | - | - | - | 1.0% (64) |
| Chronic atrial fibrillation | - | - | - | 1.0% (65) |

| | | | | |
|---|---|---|---|---|
| Non-rheumatic aortic sclerosis | - | - | - | 1.1% (69) |
| Orthostatic hypotension | - | - | - | 1.1% (68) |
| Acute cystitis | - | - | - | 1.2% (72) |
| Cerebral infarction | - | - | - | 1.1% (67) |
| Renal disorder due to type 2 diabetes mellitus | - | - | - | 1.1% (70) |
| Seropositive rheumatoid arthritis | - | - | - | 1.1% (71) |
| Complication occurring during pregnancy | - | 1.1% (128) | - | - |
| Genitourinary tract hemorrhage | - | 1.1% (128) | - | - |
| Post-inflammatory pulmonary fibrosis | - | - | - | 1.1% (67) |
| Transplanted heart valve present | - | - | - | 1.0% (64) |
| Disorientated | - | - | - | 1.0% (65) |
| Non-toxic uninodular goiter | - | - | - | 1.1% (69) |
| Acute suppurative otitis media w/o spontaneous rupture of ear drum | 1.1% (25) | - | - | - |
| Attention deficit hyperactivity disorder, combined type | 1.1% (26) | - | - | - |
| Primary insomnia | - | - | 1.0% (140) | 1.1% (69) |
| High risk pregnancy | - | 1.1% (124) | - | - |
| Peripheral venous insufficiency | - | - | - | 1.0% (63) |
| Urogenital finding | - | - | - | 1.1% (69) |
| Menopausal syndrome | - | - | 1.1% (149) | - |
| Acute bronchitis | - | - | - | 1.1% (66) |
| Kidney stone | - | - | - | 1.0% (63) |
| Unsteady when standing | - | - | - | 1.0% (65) |
| Disorder of pregnancy | - | 1.1% (120) | - | - |
| Finding of vision of eye | 1.1% (25) | - | - | - |
| End-stage renal disease | - | - | - | 1.0% (64) |
| Acute stress disorder | - | 1.0% (119) | - | - |
| Asthma | - | 1.0% (116) | - | - |
| Allergic disposition | - | 1.0% (118) | - | - |
| Disorder of intestine | - | - | - | 1.1% (67) |
| Pain in right foot | - | - | 1.0% (143) | - |
| Raised prostate specific antigen | - | - | - | 1.1% (66) |
| Tremor | - | - | - | 1.0% (63) |
